# Invasive Seaweeds in the Iberian Peninsula: A Contribution for Food Supply

**DOI:** 10.3390/md18110560

**Published:** 2020-11-16

**Authors:** Diana Pacheco, Glacio Souza Araújo, João Cotas, Rui Gaspar, João M. Neto, Leonel Pereira

**Affiliations:** 1 Department of Life Sciences, Marine and Environmental Sciences Centre (MARE), University of Coimbra, 3000-456 Coimbra, Portugal; diana.pacheco@uc.pt (D.P.); jcotas@uc.pt (J.C.); rui.miguel.m@gmail.com (R.G.); jneto@ci.uc.pt (J.M.N.); 2 Federal Institute of Education, Science and Technology of Ceará–IFCE, Campus Aracati, CE 040, km 137,1, Aracati 62800-000, Ceará, Brazil; glacio@ifce.edu.br

**Keywords:** edible seaweeds, non-indigenous seaweed species, marine invasions, nutritional value, food industry

## Abstract

The introduction of exotic organisms in marine ecosystems can lead to economic and ecological losses. Globally, seaweeds represent a significant part of these non-indigenous species (NIS), with 407 introduced algal species. Furthermore, the presence of NIS seaweeds has been reported as a major concern worldwide since the patterns of their potential invasion mechanisms and vectors are not yet fully understood. Currently, in the Iberian Peninsula, around 50 NIS seaweeds have been recorded. Some of these are also considered invasive due to their overgrowth characteristic and competition with other species. However, invasive seaweeds are suitable for industrial applications due to their high feedstock. Hence, seaweeds’ historical use in daily food diet, allied to research findings, showed that macroalgae are a source of nutrients and bioactive compounds with nutraceutical properties. The main goal of this review is to evaluate the records of NIS seaweeds in the Iberian Peninsula and critically analyze the potential of invasive seaweeds application in the food industry.

## 1. Introduction

Seaweeds’ ecological relevance has been acknowledged by the scientific community through the assessment of the ecosystem services they provide, which directly or indirectly support human well-being, namely regulating, provisioning, and cultural services [1,2].

Algae play a pivotal regulatory role in the aquatic environment, being sources of primary and secondary production, providing protection to coastal zones, and as nursery areas [3]. Moreover, seaweeds are a food source for many aquatic organisms, supporting provisioning services for a wide range of invertebrates [4]. Furthermore, seaweeds are part of cultural heritage and distinctiveness in each area, presenting economic value for society [5].

However, these ecosystems are currently under threat due to climatic changes, such as ocean acidification or the increasing seawater temperature [6]. Despite that, anthropogenic pollution nodes are major stressors that affect the structure and functioning of aquatic environments. For instance, eutrophication is a phenomenon provoked by the discharge of effluents with a high inorganic load (i.e., phosphorus, nitrogen, nitrate, nitrite) that can lead to eutrophication. Thus, the excess of nutrients will lead to the occurrence of algal blooms, giving an advantage to opportunistic algae that will affect the structure of the community and its primary productivity [7]. Regarding the available resources, non-indigenous species (NIS) usually are more effective when using them than native species [8]. However, NIS presence can have negative, positive, or neutral effects on the ecosystems where they are integrated [9]. Still, the introduction of exotic species in marine ecosystems can often lead to severe changes in ecosystem functioning. Among the vectors which favored the introduction of exotic seaweeds related to marine traffic rising [10] are, namely, through the discharge of ballast waters and biofouling on recreational boats or cargo ships hulls [11,12,13]. Another possible introduction vector is associated with the aquaculture, importation, and commercialization of marine organisms, namely mollusks [14]. Moreover, it is still found exotic and/or invasive species commercially available in the European aquarium trade markets, such as *Caulerpa racemosa* and *Caulerpa taxifolia* [15].

Whenever exotic seaweed species are introduced in a different geographic area and if the biotic and abiotic conditions allow it, these species can exhibit an invasive behavior. Moreover, a set of characteristics are needed to consider it an invasive seaweed species, commonly related to opportunistic traits such, such as a fast growth rate, dynamic life cycle, high recruitment rate, physiology, size, and fitness [3,16,17,18]. However, the intrinsic mechanisms associated with the biologic invasion’s success are not yet fully understood due to their complexity [3]. For these reasons, macroalgal marine invaders are considered a threat to coastal and estuarine environments. 

The Iberian Peninsula macroalgal community has also been a target of the introduction of several exotic seaweed species (Table 1), in which some of them are well established, exhibiting a widespread distribution in this area and invasive behavior.

Against this scenario, it urges the need to mitigate the impacts of seaweeds invasions in coastal and estuarine ecosystems, prioritizing the mapping of exotic seaweed species and comprehend the mechanisms which allow the success or failure of the invasion in order to take control and conservation measures [17,28,29]. 

Among the several adaptative advantages of invasive seaweeds, their high growth rate makes them suitable to feedstock supply for industrial exploitation. Thus, the goal of this review is to critically analyze the potential of invasive seaweeds’ direct or indirect application in the food industry.

## 2. Invasive Seaweeds: An Important Feedstock to Food Industry

### 2.1. Red Seaweeds

**Figure 1:**
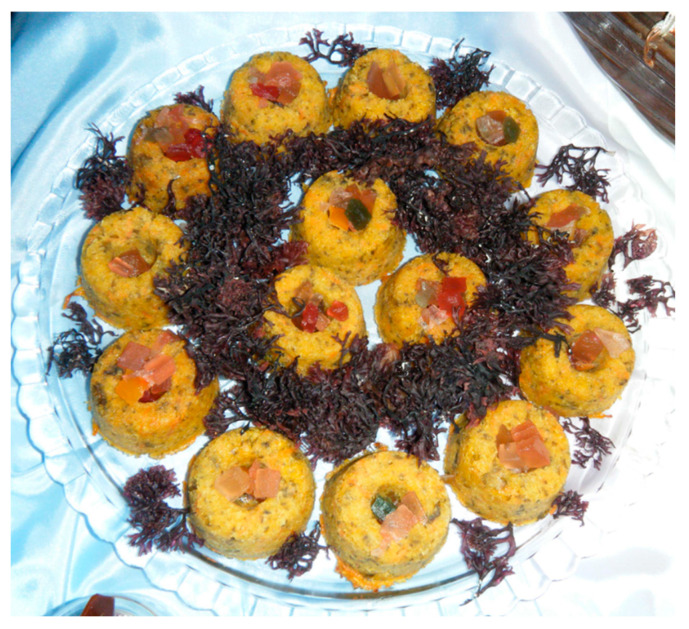
Seaweed (*Pyropia* sp.) pie with carrot and coconut.

In marine ecosystems, seaweeds that belong to the phylum Rhodophyta constitute a wide taxonomic diversity [30]. Among the exotic seaweeds registered on the Iberian Peninsula, *Pyropia suborbiculata* (Kjellman) (J.E. Sutherland, H.G. Choi, M.S. Hwang and W.A. Nelson 2011 it is an Asiatic Bangiales), due to its high tolerance to the variation of physico-chemical conditions, is now widespread throughout the American, Australian, and European shoreline [31,32,33]. However, the first records of *P. suborbiculata* in the Atlantic Ocean were misidentified with *Neopyropia yezoensis* M.S. Hwang and H.G. Choi (formerly *Pyropia yezoensis*) [34]. Through molecular analysis, in 2005, it was possible to genetically distinguish these two seaweed species [35]. More recently, researchers found that *P. suborbiculata* is well established on the Iberian Peninsula and is genetically similar to the population from the Pacific Ocean, suggesting that the presence of this exotic seaweed in the Northwest Atlantic is probably through marine shipping [31,36]. In contrast, *P. suborbiculata* is produced through aquaculture and is authorized and considered safe for human consumption in the United States of America [37,38]. In fact, this seaweed can have a significant role in the daily diet (Figure 1) and can be used fresh or dried, milled, and then utilized as a flavor enhancer [39].

However, it is in the Asiatic region that this seaweed currently assumes a high economic interest, being considered an important marine crop for food feedstock [40,41]. In these countries, seaweeds which belongs to *Pyropia*/*Porphyra/Neopyropia* genus are highly consumed by the population [42]. Therefore, *P. suborbiculata* is also a potential candidate for food industry feedstock due to its protein (11.2% DW), lipids (0.3% DW), and carbohydrates (31.6% DW) content [43]. 

The health benefits that this food resource presents, lead to increased customer demand, which allowed a sales volume increase and the global economic expansion of *Pyropia*/*Porphyra* commercialization [44].

Thus, *Pyropia*/*Porphyra/Neopyropia* spp. farming became essential to guarantee the feedstock. However, these cultivations are frequently affected by fungal diseases. For instance, the fungi *Pythium porphyrae* provokes the most concerning disease (red rot disease) in Asiatic *Pyropia* aquacultures [45,46], causing seaweed blades destruction, precluding the entire cultivation, and leading to serious economic losses [47]. Nevertheless, studies showed that *P. suborbiculata* is more resistant to *P*. *porphyrae* fungal attack [48], thus being a potential candidate for food supply through their cultivation. 

**Figure 2:**
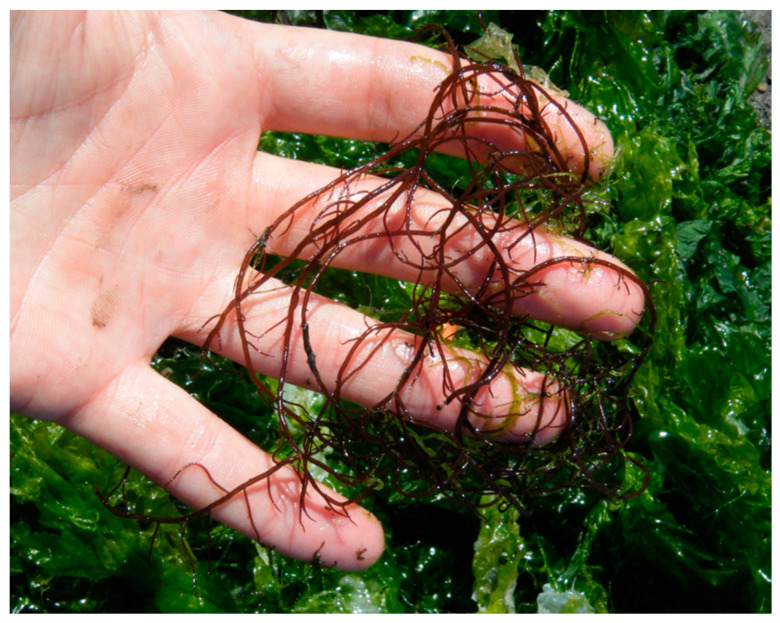
*Agarophyton vermiculophyllum* collected in an aquaculture fish tank at Ria de Aveiro (Portugal).

Another introduced red seaweed native from Japan, *Agarophyton vermiculophyllum* (Ohmi) Gurgel, J.N. Norris et Fredericq 2018 (previously known as *Gracilaria vermiculophylla*) (Figure 2), has invaded estuaries throughout the whole world. Although the presence of this algae was underrated in several areas due to the morphological similarity with native species *Gracilaria gracilis* [49], many signs of progress have been made through genetic analysis in order to distinguish them [50,51]. 

In Europe, some authors defend that this seaweed was unintentionally introduced through Japanese oysters farming, migrating birds, or shipping [50]. Since then, this species is well established in the Iberian Peninsula because they can tolerate abiotic parameters variation such as temperature (11–25 °C) and salinity (10–30 PSU) [50]. Due to this species resilience, they are recognized by their environmental and ecological impact on fauna and flora [4,52,53].

From a nutritional perspective, *A*. *vermiculophyllum* is rich in monounsaturated and polyunsaturated fatty acids (22.2 mg/kg DW and 4 mg/kg DW, respectively) [54]. They also have interesting phosphorus (0.082–0.203%) and nitrogen (2.27–4.68%) content [55]. 

For this reason, this seaweed already had shown to be a low-cost tool to supplement animal feed to improve aquaculture fish nutritional profile and their organoleptic quality. For instance, researchers found out that adding only 5% of *A*. *vermiculophyllum* on rainbow trout feed increase fish iodine levels and improve fillet color and texture [56].

However, this agarophyte seaweed also indirectly contributes to the food industry. Agar is a phycocolloid highly valuable for this sector. In this red seaweed, agar content can reach up to 30% of their dry weight [57] and it could be incorporated in food products as gelling, stabilizing, and encapsulating agent due to its rheological properties [58,59]. Furthermore, agar biofilm enriched with *A*. *vermiculophyllum* extract can be used in edible fruits and vegetables, maintaining properties such as color and light gloss up [58].

More recently, aiming for plastic waste reduction, *A*. *vermiculophyllum* was used to develop sustainable fish packaging [60]. In this study, the extracts from this seaweed were applied to allow the antimicrobial activity of the fish packaging [60] Furthermore, seaweeds can contribute to innovation in the food sector while contributing to food security [61].

**Figure 3:**
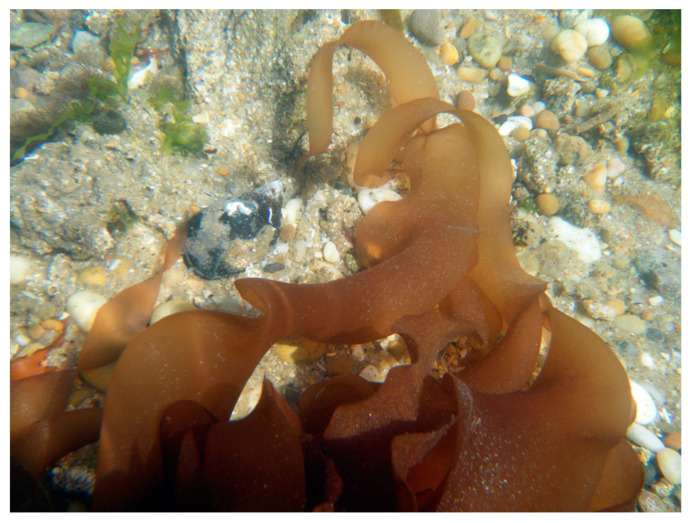
Underwater photography of *Grateloupia turuturu* in Buarcos Bay (Figueira da Foz, Portugal).

*Grateloupia turuturu* Yamada 1941 (Figure 3) shows a resilient behavior, presenting a high reproduction rate and tolerating a wide range of temperatures (4–29 °C) and salinity (22–37 PSU), thus threatening several native seaweeds [62]. Therefore, this seaweed native from Japan currently exhibits a cosmopolitan distributional pattern [14]. However, until 2002, the taxonomic identification of *G. turuturu* was misidentified with *Grateloupia doryphora* (Montagne), whereas a research group was able to distinguish them through molecular techniques [63]. Hence, the first occurrence of this seaweed in Europe was in 1982 and was observed in the Iberian Peninsula in the early 1990s [64]. The authors hypothesize that this species was introduced through their biofouling capacity on hulls or oyster shells [14].

Despite the records of the direct consumption of *G. turuturu* in Asian countries [65], the full nutraceutical potential of this seaweed is still unrevealed in Europe. However, some researchers are focused on their chemical composition and their bioactivities. *G. turuturu* is an edible seaweed that already contributes to food demand due to their protein (22% DW), lipid (2% DW), dietary (60% DW), insoluble fibers (12% DW), and sterols content [65,66,67,68]. It is also important to highlight the concentration of phycoerythrin (0.30% DW) and phycocyanin (0.033% DW) [67]. Besides that, this macroalgae is also a source of essential amino acids such as histidine (1.8 g protein-N), leucine (6.3 g protein-N), tryptophan (0.7 g protein-N), lysine (4.3 g protein–N), methionine (2 g protein-N), phenylalanine (3.7 g protein-N), threonine (3 g protein-N), and valine (4.9 g protein-N) [66,69].

Researchers evaluated the nutritional profile of this red seaweed in Portugal [70] (Table 2) and demonstrated that the most abundant macronutrients are sodium (Na) and potassium (K), exhibiting 96.08 and 20 mg/g DW, respectively. While magnesium (Mg), calcium (Ca), and phosphorus (P) were found at a concentration ranging 2–2.81 mg/g DW. Regarding the micronutrient concentration, most representatives were zinc (Zn) and iron (Fe), while the other trace elements analyzed presented a vestigial concentration (Table 3). 

In fact, the daily intake of these elements is extremely important to the human body and guarantees good metabolic functions [71]. For instance, a study conducted by Pang et al. (2006) [72] with *G*. *turuturu* collected in China showed that this edible red seaweed can be a human health promoter through their antibacterial activity against *Vibrio parahaemolyticus*. 

**Figure 4:**
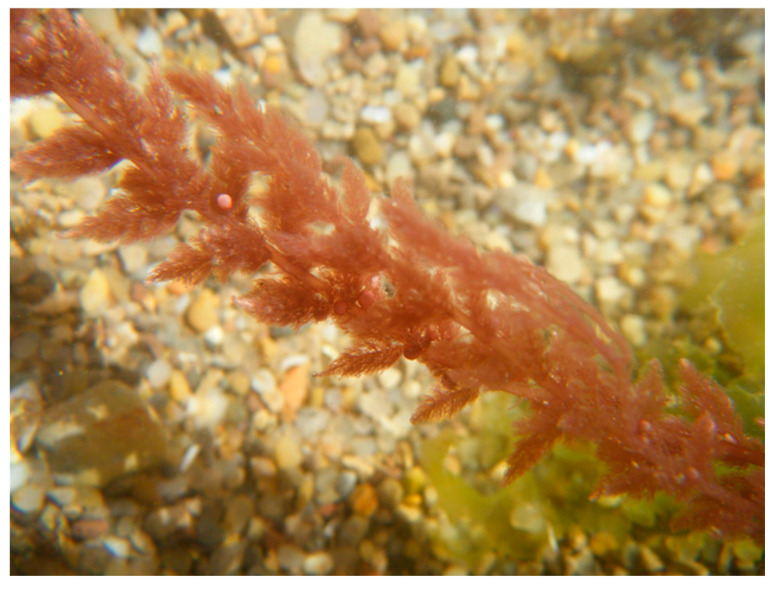
Underwater photo of *Asparagopsis armata* in São Martinho do Porto (Portugal).

The red seaweed *Asparagopsis armata* Harvey 1855 (Figure 4), native from Australia, was intentionally introduced in Europe due to the high food demand in 1920 [78,79,80]. Then, this seaweed species was maintained by aquaculture in Ireland [79], which lead to their dispersion through this country. 

Some authors hypothesize that the introduction of exotic seaweed in the Iberian Peninsula was associated with oyster transportation and commercialization [81,82]. Since then, this seaweed species has been well established in the Northwest coast of the Iberian Peninsula [83].

There are records that this species has been used as food [84]. In fact, *A. armata* is rich in several micronutrients (Table 3) that are essential in lower concentrations to the good function of the human body, such as calcium (4.47% DW), sodium (9.36% DW), magnesium (1.38% DW), and phosphorus (0.27% DW). However, this seaweed is also rich in trace elements that are important in lower concentrations to human health, namely zinc (66.3 mg/kg), copper (13 mg/kg), manganese (62.3 mg/kg), and iron (1188 mg/kg) [70,77]. Moreover, *A. armata* contains a high protein content that can reach 18.3% DW, with the synthesis of essential amino acids, such as isoleucine, valine, lysine, methionine, phenylalanine, histidine, and tryptophan, which the human metabolism is not able to synthesize [85]. 

Besides that, the chemical composition of this seaweed presents several interesting bioactive compounds with applications in the food industry. In the Portuguese coast, researchers found that sterols represent 555 mg/kg *A*. *armata* dry weight namely, desmosterol, fucosterol, and b-sitosterol [86,87], which are anti-cholesterol compounds [87,88]. Moreover, this red alga also contains halogenated metabolites, such as bromine, chlorine, and iodine-containing methane, ethane, ethanol, acetaldehyde, acetone, 2-acetoxypropane, propene, epoxypropane, acrolein, and butenone [89,90,91,92], which are bioactive molecules with antifungal, antimicrobial and antibiotic effects [90,93,94]. 

Hence, the direct consumption of this seaweed, even in lower quantities, can be considered a supplement to the human daily diet. However, many studies have currently been focused on the incorporation of *A*. *armata* as an animal feed supplement in order to improve meat quality and reduce methane emissions [95].

**Figure 5:**
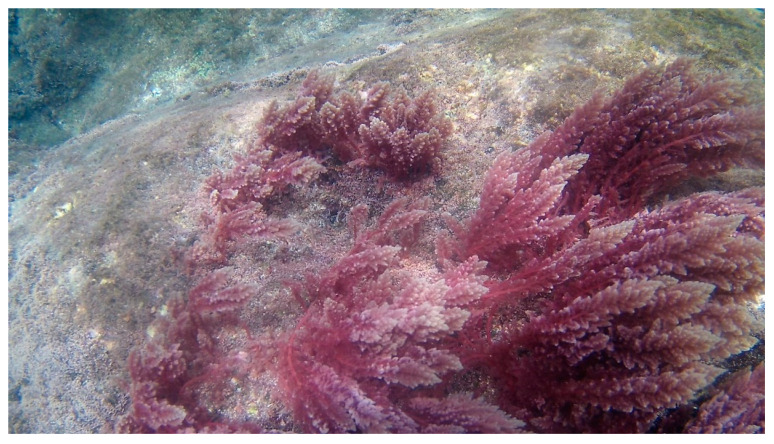
Underwater photo of *Asparagopsis taxiformis* in Terceira island (Azores archipelago -Portugal).

*Asparagopsis taxiformis* (Delile) Trevisan 1845 (Figure 5) is a red seaweed native from Australia [96], with a high capability to cope with temperature variations [97], thus being distributed through tropic and sub-tropical regions [98]. This species is currently well established in Europe and is considered an invasive seaweed species in the Iberian Peninsula, particularly in Spain and in the Portuguese archipelagos (Azores and Madeira), due to their coverage area and noxious effects on the surrounding fauna and flora [74,98,99].

Among other seaweeds, there are historical records in Hawaii of *A. taxiformis* usage as a nutraceutical food due to their lipids, proteins, and carbohydrates (Table 2) content [100]. Further studies corroborated the ethnobotanical application of this seaweed in the Hawaiian daily diet, revealing the biochemical composition of this seaweed. 

In the Portuguese islands, the biomass of this seaweed was also analyzed in order to evaluate its nutritional profile, revealing to be a good source of macronutrients [73]. For this reason, many recipes including *A. taxiformis* were developed, such as soups, salads, or scrambled eggs [101].

*Asparagopsis taxiformis* presents a rich composition in micronutrients and trace elements (Table 3). For instance, Selmi et al. (2020) [76] evaluated *A. taxiformis* nutritional profile of biomass harvested from Tunisia, which revealed a high content in iron (0.2189 mg/g), sodium (0.200 mg/g), and potassium (0.13784 mg/g), which are pivotal micronutrients to the good function of osmoregulatory processes in the human body. Meanwhile, the trace element with more representativity in *A. taxiformis* biomass was manganese, with a concentration of 3.05 × 10^−3^ mg/g. However, the other elements, namely, arsenic, cadmium, copper, mercury, and lead, demonstrated to be present in concentrations lower than 5 × 10^−4^ mg/g. 

This seaweed also presents nutraceutical potential as an iodine supplier, presenting a concentration up to 3.37 g/100g DW, benefiting people who suffer from a deficit of this micronutrient [102,103].

In summary, the invasive seaweeds from the Iberian Peninsula analyzed exhibit a rich nutritional composition in macro and micronutrients that are pivotal to complement a human health diet, even in low amounts (Table 3). However, it is necessary to consider that seaweed nutritional profiles vary according to the species, geographical place, tidal exposure, season, physico-chemical composition of the water, or even with the seaweed processing techniques [104]. Regarding the direct seaweed application in the food industry, consumers hold a concern relatively to metal concentration. Table 3 shows the nutrient value reference (NVR) according to the European Food Safety Authority (EFSA) [105]. Researchers analyzed *A. taxiformis* content in warning pollutants, namely As (4 × 10^−4^ mg/g DW), Cd (2 × 10^−5^ mg/g DW), Hg (2 × 10^−5^ mg/g DW), and Pb (5.1 × 10^−4^ mg/g DW), revealing lower concentrations relative to the NVR for each element [106,107,108,109,110].

### 2.2. Brown Seaweeds

**Figure 6:**
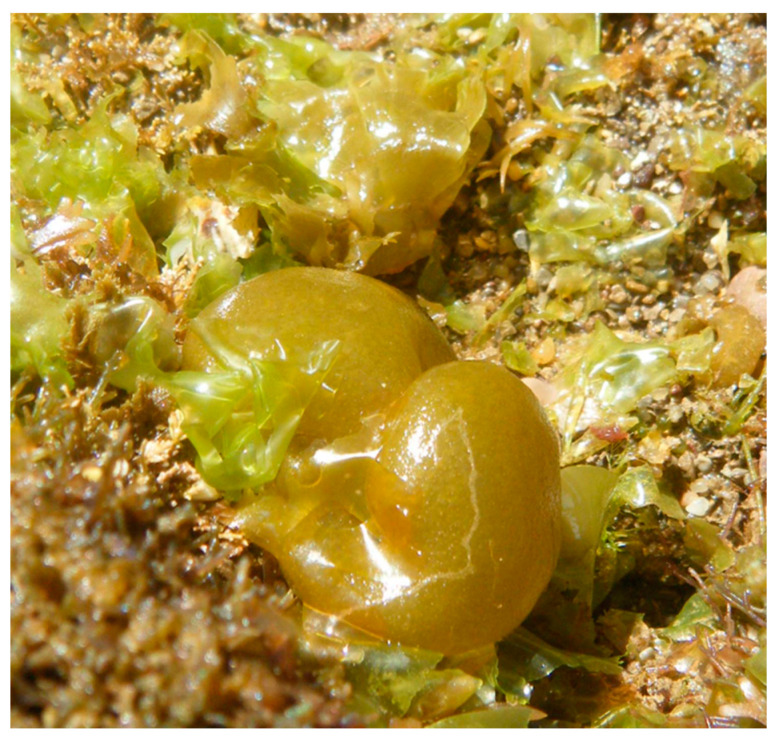
*Colpomenia peregrina* in São Martinho do Porto (Portugal).

*Colpomenia peregrina* Sauvageau 1927 (Figure 6) is a brown seaweed native from the Northwest Pacific [111]. This species is characterized for being annual, with globular physiology and for being extremely tolerant to environmental conditions variation, such as salinity (15–30 PSU) and temperature (13–20 °C) [112,113]. For this reason, *C*. *peregrina* is such a cosmopolitan species with ease in establishing in new areas. However, this exotic species can be easily misidentified with a native Portuguese seaweed, *Colpomenia sinuosa*. Thus, *C. peregrina* is characterized by the thinness of the thallus, hairs arising from the sub-cortical cells not associated with the sori, which are confluent, not punctate, and have no pellicle over the plurilocular sporangia, which are shorter than those of *C*. *sinuosa* [114].

This exotic seaweed was firstly observed in Cadiz (Spain) in 1806 [115] and nowadays is present in the European temperate regions, including the Northwest of the Iberian Peninsula [116]. Researchers hypothesize that the introduction of this NIS could have occurred through the coastal cultivation of oysters in France. This seaweed grows attached to the oyster shells and when *C. peregrina* bladder fills up with air and water, is transported through the oceanic streams [116,117]. 

Despite the widespread distribution of *C. peregrina*, there are just a few studies regarding their nutritional profile and biomass valorization. Nevertheless, a group of researchers harvested this species in Southwest England in the United Kingdom (U.K.) and determined that the composition of *C. peregrina* is mostly minerals, representing 85.3% of this seaweed dry weight, in which 12.2% DW are carbohydrates, 2.48% DW protein, and 0.8% DW lipids [118]. Further analysis was performed in order to evaluate their micronutrient profile (Table 4).

According to the available data, the high K and P content (46.93 and 0.67 mg/g, respectively) indicates that this seaweed can be a potential biomass feedstock for the agriculture industry as a natural fertilizer. Regarding as a food source, *C. peregrina* is valuable due to its Ca content (55.64 mg/g), which can be beneficial for people who suffer from diseases related to calcium deficit, such as osteoporosis [119].

Despite the high silicon content (252.29 mg/g) [118], it was estimated that a daily dietary intake of 20–50 mg silicon/day by an average adult with 60 kg is unlikely to cause health problems [120]. Nevertheless, it is necessary to consider that the aluminum concentration found in *C. peregrina* exceeds the tolerable upper intake recommended by EFSA. 

**Figure 7:**
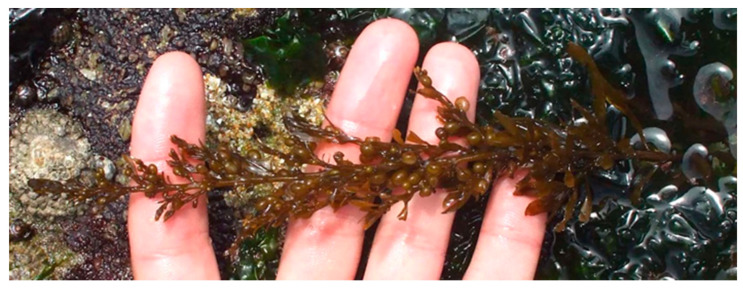
*Sargassum muticum* in Buarcos Bay (Figueira da Foz, Portugal).

*Sargassum muticum* (Yendo) Fensholt 1955 (Figure 7) is a native seaweed from Japan and China, being currently widespread along the European shoreline. Due to their high tolerance to hydrodynamic, temperature, and solar exposure variations, this species possess a huge ability to acclimatize and maintenance in different areas with distinct climate conditions [121,122]. Hence, their fast reproduction and growth rate makes them thrive after their introduction, assuming an invasive character [123,124]. 

These Japanese-native seaweed species was inadvertently introduced into European Atlantic waters in the early 1970s. The first record of *S. muticum* in Europe was in the English Channel [125] and quickly spread to the Iberian Peninsula [126,127,128]. It is necessary to consider that the presence of this seaweed in marine ecosystems causes a serious macroalgal biodiversity loss due to the shading they produce, blocking the sunlight to other native species [129,130,131]. The authors theorize that the introduction of *S. muticum* in the Iberian Peninsula was probably due to oysters for aquaculture or through the spread of spores during transport of ballast water and its discharge [131].

Seaweeds chemical characterization mostly reflects the abiotic and biotic conditions where they grow, so according to the harvesting area, *S. muticum* biomass will present variations in the nutritional profile, namely in the macronutrients (Table 5), micronutrients, and trace elements (Table 6). 

In general, *S. muticum* nutritional profile from India, whereas the fresh biomass was analyzed, is notably lower than the macroalgal biomass collected in the other countries.

Comparatively, with the biomass collected in Spain, *S. muticum* harvested in the U.K. presented higher carbohydrates and ash content, which could possibly be related to the chemical composition presented in the marine ecosystem. In contrast, the lipid concentration of the biomass harvested in the U.K. and Spain exhibited a similar value. For instance, *S. muticum* collected in Spain had a variation between 20.10% and 31.77% of total fatty acids content, whereas polyunsaturated fatty acids composition during the year presents a balanced ω-6/ω-3 ratio (1.40–3.37) [133], which is the recommended ratio for human food consumption by the World Health Organization (WHO) and as reviewed by Leandro et al., 2020 [61].

The overall biochemical analysis also shows that *S. muticum* contains a high concentration of carbohydrates, mostly alginic acid, which has a behavior of dietary fiber in the human gastric system.

However, it is also necessary to consider that the chemical composition of the macroalgal biomass varies within the biotic and abiotic parameters fluctuation in each sampling site [43]. Besides that, the drying process (time and temperature) can interfere with the nutritional yield and further valorization [133]. 

The nutrient richness of this seaweed species is highlighted in Table 6. Even though seaweed species are well-known metal bioaccumulators, the metal content analyzed in the biomass harvested in Spain exhibits metal concentrations below the threshold defined by EFSA [105,108,120].

Regarding the differences observed within the countries analyzed, is necessary to consider that seaweeds’ chemical composition can be affected by several factors, such as the nutrient’s availability, pollution nodes, and other environmental parameters related to the geographical zone where the biomass is harvested [134,135]. However, this can only be corroborated with previous physico-chemical characterization of the habitat. 

Although *S. muticum* has been already exploited for aquaculture production in China [136] and it is a traditional food in Korea [137], there is no commercial use of this biomass for food consumption in Europe nor in rest of the non-native location [138]. 

*S. muticum* contribution as an value-added product has been reviewed by Milledge, Nielsen, and Bailey (2016), highlighting the application as food, feed, and biofertilizers to agriculture crops [139].

Silva et al. (2019) [140] analyzed the effects of aqueous extracts of *S. muticum* as seed germination enhancer and as plant biofertilizer in two commercial sub-variants of *Lactuca sativa* (Lettuce) [140]. However, the germination assay proved that a high seaweed extract concentration inhibits seed germination. Nevertheless, the concentration of 25% was the best concentration for seed germination. In the biofertilizer assay, the same extract concentration (25%) demonstrated to be the most effective treatment. The *S. muticum* extract treatment revealed also that the plants were able to assimilate a higher minerals content, mainly P, K, Ca, Mg from the soil. These results show that *S. muticum* treatment can have an efficient multirole effect in the agricultural crop.

Regarding the animal feed industry, *S. muticum* can also contribute as a feed supplement to the holothurian and abalone aquaculture cultivation [141]. 

**Figure 8:**
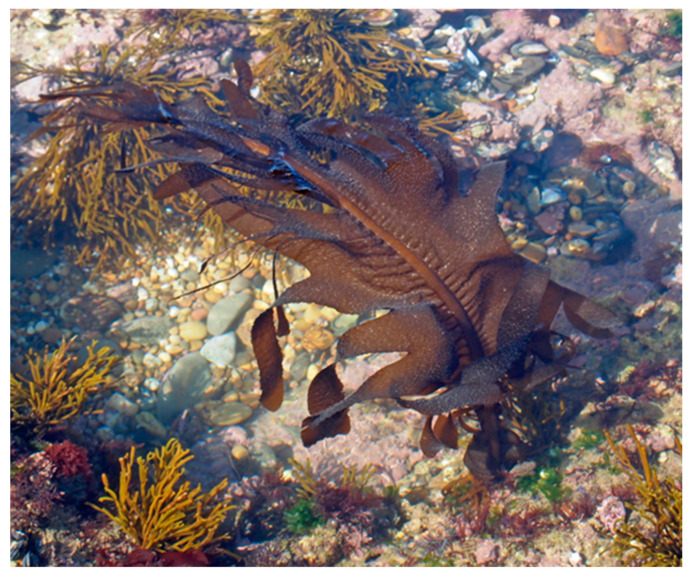
*Undaria pinnatifida* in Buarcos Bay (Portugal).

*Undaria pinnatifida* (Harvey) Suringar 1873 (Figure 8) is annual kelp, native from the Northeast Pacific, being present in the shoreline of different countries such as Japan, China, Korea, and Oriental Russia. This species can stand a wide range of environmental conditions, such as temperatures from 0–27 °C and salinities as low as 20 PSU [142]. For this reason, this species is nowadays well established in several locations, namely, in the Atlantic, Mediterranean Sea, Pacific Oceania, and Atlantic America coastline, from Argentina to the United States of America [143].

*Undaria pinnatifida* was first registered in European shoreline in France in 1971 [144]. A probable introduction vector of this Asiatic species into the Iberian Peninsula can be related to the transportation of Pacific oysters, in resemblance with other exotic seaweed species [145]. The two other possible vectors responsible for the introduction of *U. pinnatifida* are mainly considered to be by boats (mainly trans-Atlantic boats, with cargo boats being one of the best examples for the accidental introduction of NIS); however, accidental introduction by aquaculture is also very well documented, mainly in 18 and 19 centuries [146,147]. Their ecological impact on native fauna and flora is even more concerning than other exotic seaweed species, considering that this is an annual species [148,149]. 

However, the harvesting and exploitation of this seaweed for direct food consumption, mainly in the Asiatic countries, already control *U. pinnatifida* biomass and is a sustainable way to mitigate the ecological disturbances caused by their presence in the ecosystems [150].

In Spain, *U. pinnatifida* is collected and afterward is usually dried in order to be sold for direct consumption [151] or as an ingredient in pre-cooked meals [152]. So, this invasive species cannot be cultivated in non-native habitats but can be harvested from coastal areas and incorporated into the daily human diet, direct or indirectly. Due to the high exploitation of this edible seaweed worldwide, the information available regarding the food nutritional values is very extensive, unlike the other exotic seaweeds analyzed. It is shown how the macronutrients (Table 7), micronutrients, and trace elements (Table 8) concentration varies according to the harvesting site. 

According to the literature, *U. pinnatifida* nutritional profile does not suffer many fluctuations between geographical zones, presenting identical results regarding lipids, proteins, carbohydrates, fibers, and mineral content. However, the lipid and protein profile could be different or exhibit different concentrations of each compound [153,155]. Moreover, *U. pinnatifida* synthesizes essential amino acids such as histidine, isoleucine, lysine, valine, phenylalanine, and methionine, but in different concentrations according to the geographical region [153,155]. Furthermore, *U. pinnatifida* from both sites also demonstrated low variations in their macronutrient concentration. 

From another perspective, the micronutrient and trace metals concentration are directly affected according to the geographical harvesting zone (Table 8). According to Taboada et al. (2013) and Kolb et al. (2004) [153,155], *U. pinnatifida* revealed similar contents of potassium in both geographical areas (Japan and Spain). Comparatively, *U. pinnatifida* biomass harvested in the native zone presented a higher content on calcium and sodium, while the biomass harvested from the Northwest Iberian Peninsula contained higher concentrations of phosphorus and magnesium. 

The biomass collected in the invasive place showed a higher concentration in all the presented trace elements, except for nickel and chromium, for which the authors did not present information regarding the concentration of these trace metals in *U. pinnatifida* from Spain. 

Researchers performed the analysis of the iodine (I) content in commercial samples from Europe and from Japan, whereas the European samples revealed a higher I concentration [156]. Thus, the recommended daily intake dose will be different, according to the origin of the seaweed [61].

It is proven that food consumption behavior can modify the skeletal muscle mass and performance. Extracts from the brown seaweed *U. pinnatifida* were tested on mice as a potential candidate to develop a product to promote muscle mass and functioning [157]. These extracts enhanced the mitochondrial biogenesis, which consequently increased the oxidative muscle fiber in mice and stimulated the angiogenesis in skeletal muscles. Thus, enhanced the mice’s exercise capacity and skeletal muscle mass. The observed effects were derived from the presence of fucoxanthin, hesperetin, and caffeic acid and other bioactive molecules detected in *U. pinnatifida* extracts.

*Undaria pinnatifida* fatty acids demonstrated antioxidant and antibacterial activity, which can be used in the food industry [158]. Moreover, several studies have been focused on the application of bioactive compounds extracted from this brown seaweed, such as pigments or polysaccharides as flavor enhancers and to improve the organoleptic properties of food products [135,159].

However, this seaweed can also contribute indirectly to the food industry through crop culture. In fact, this seaweed was already tested as a soil amendment in poor quality soils in tomato production. The results obtained benefited the soil properties and promoted a multirole action as a soil fertilizing agent and enriching the plant with phytohormones and vitamins [160]. 

### 2.3. Green Seaweeds

**Figure 9:**
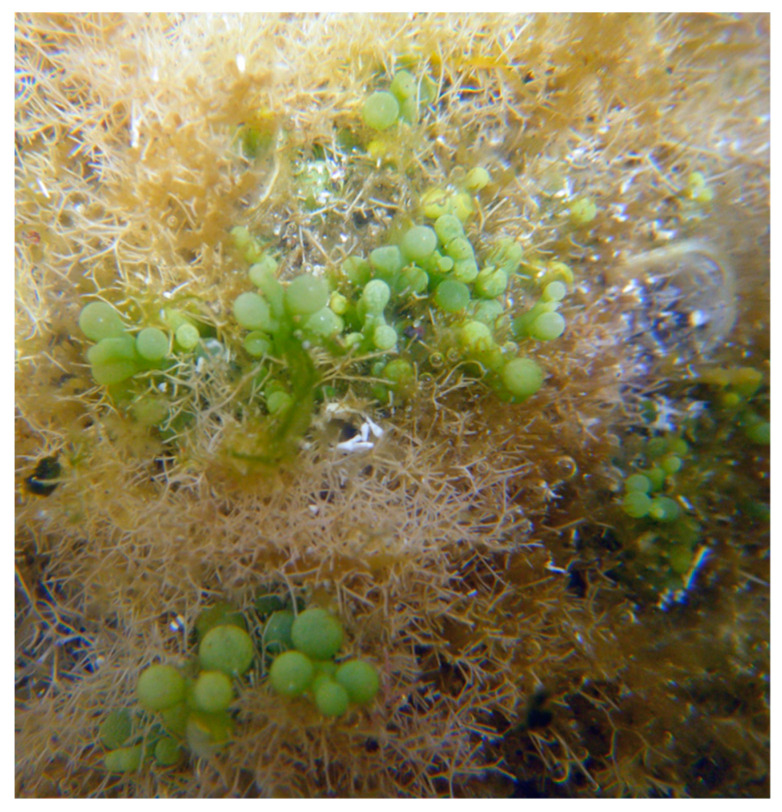
Underwater photography of *C*. *racemosa* in the Spanish islands.

The green seaweed *Caulerpa racemosa* Forsskål) J. Agardh 1873 (Figure 9) is native from Australia [161]. The presence of this seaweed leads to a significant biodiversity loss, especially in invertebrate diversity and abundance [161]. This seaweed inhabits tropical and warm-temperate regions but tolerates low temperatures down to 10 °C, being a resilient species that easily adapts to variations of biotic and abiotic parameters [161]. For this reason, *C. racemosa* has currently a widespread distribution throughout the world [162,163,164]. In Spain, the existence of the invading *C*. *racemosa* in Alicante, at a depth of 0 to 2 m, was reported [165]. There are several hypotheses to this seaweed species introduction in the Iberian Peninsula, namely, the unintentional introduction through ballast waters and sediment, ship hull fouling, or the through the trade of aquarium species [161]. 

There are records of the use of *C. racemosa* in the Indo-Pacific region, where it is appreciated due to the taste, texture, and health-promoting bioactivities [166]. In fact, this species contains an interesting macronutrient composition, namely, in protein (0.6–18.3% DW), carbohydrates (3.6–83.2% DW), lipids (0.1–3.8 8% DW), moisture (8.8–91.5% FW), and ash (14.5–55.1% DW) [167,168,169]. 

Moreover, *C. racemosa* contains a micronutrient and trace elements (Table 9) profile based on iron 29.71%, sodium 10.64%, zinc 6.82%, potassium 5.03%, manganese 4.91%, calcium 4.76%, magnesium 1.61%, copper 0.62%, molybdenum 0.15%, and selenium 0.13% [170].

Fractions of sulfated polysaccharides isolated from the extract of the *C. racemosa* of the coast of Gujrat (India) were analyzed for sugar content and galactose, glucose, arabinose, and xylose were found as the main components [171]. The macroalgae have, among other purposes, the presence of polysaccharides with anti-herpetic activities according to the authors. In addition, they have anti-nociceptive and anti-inflammatory activities [172]. Recently, Reference [173] reported that silver nanoparticles mediated by the macroalga *C. racemosa* showed excellent antibacterial activities against human pathogenic bacteria *Staphylococcus aureus* and *Proteus mirabilis*. In one study, the antioxidant and phenolic content of *C. racemosa* was higher than red seaweeds [174].

The macroalgae *Caulerpa taxifolia* (M. Vahl) C. Agardh (1817), native from the tropical and subtropical region of Australia, specifically in Moreton Bay, Southern Queensland, and Lord Howe Island [175], was the first macrophyte invasion to attract public attention. Consequently, authorities in some Mediterranean countries, such as Spain and France, tried to eradicate and control this invasion [144]. According to the authors, the two *Caulerpa* species were introduced and can co-occur in certain areas of the Mediterranean Sea, such as in Italy, France, Croatia, and Spain. In terms of biomass, *C. taxifolia* is more prominent than *C. racemosa* [176].

Thibaut, Meinesz, and Coquillard (2004) clearly showed that one of the main characteristics of the *C. taxifolia* macroalgae is its perennial life cycle, with relatively high biomass values throughout the year, in different biotopes, which may be a factor in the broad ecological impact of this species on the aquatic environment [177].

This macroalga can present up to 13% of toxic metabolites, which makes its consumption by herbivorous organisms unpleasant [178,179].

For instance, caulerpenyne is the main secondary metabolite produced by *C. taxifolia* and it plays an important role in the chemical defense of algae [178,179,180]. The presence of this invasive species reduces the availability of microhabitat for fish, resulting in a decrease in species richness, density, and biomass when compared to native communities that live in beds or rocky areas [181]. This metabolite has several biological activities, mainly antitumor activities inhibiting the growth of several cancer cell lines [182]

*Codium fragile* ssp. *fragile* (Suringar) Hariot 1889 is apparently originated in the Pacific Ocean, more specifically from the coast of Japan [183], and is one of the most invasive species in the world, having spread from its native range in the NW Pacific to the NE and SE Pacific, N Atlantic, Mediterranean, and the coasts of South Africa, Australia, and New Zealand [184,185].

Once established, *C. fragile* subsp. *fragile* grows rapidly and has an inhibitory effect on the recruitment and growth of kelps [186,187]. This resulted in the replacement of *Laminaria* beds by *C. fragile* meadows in the Gulf of Maine and along the Atlantic coast of New Scotia [188].

Watanabe, Targets, and Scheibling (2009) [189] cited that the wide variety of propagules are produced by *C. fragile* and the variable distances over which different types of propagules can be transported give this species the advantage of short- and long-distance dispersion, and probably played a role in the invasive success of this alga in various aquatic environments [189]. This macroalga can be limited by nitrogen availability and its growth and propagation is related to the concentration of nutrients in seawater (for example, eutrophication and resurgence events) [184]

Regarding the fatty acid content, researchers analyzed six species of *Codium* in southwestern Australia and found that for the native *C*. *fragile* alga, the largest amount was of hexadecanoic acid (16:0), 40.50%, followed by oleic acid (18:1ω9), 23.30%, linolenic acid (18:3ω3), 5.60%, and myristic acid (14: 0), 5.30%, also presenting 21.10% of lipids [190]. Herbreteau et al. (1997) [191] collected this macroalga on the Sillon Bay, Cotes d’Amor, France, and obtained yields of decanoic acid (10:0) 48.60%, hexadecanoic acid (16:0) 19.70%, octadecatrienoic acid (18:3) 12.20%, and octadecenoic acid (18:1) 6.20% [191].

Regarding the sulfated polysaccharides in *C. fragile* collected on the coast of PuTian, China, extracting for 3 h at 115 °CS obtained a yield of 2.10%. In addition, they obtained yields of 30.9% of total sugar, 4.05% of uronic acid, 39.7% of sulfate, and 282 kDa of molecular weight [192].

Rojo et al. (2014) [193] observed that on the Cantabrian coast, northwestern Spain, the invasive *C. fragile* matured in a smaller size and that this could confer competitive advantages over the native species of the habitat.

To the west of the Iberian Peninsula, in Spain, [29] the effectiveness of marine protection in containing the invasion of six marine macroalgae was evaluated, including *Codium fragile*, and it was found that the presence of this species was determined by the interaction between exposure and protection, indicating significantly greater biomass in locations outside Marine Protection Areas (MPAs) compared to those within the MPAs. The authors cite that the protection provided by MPAs is very limited to prevent the establishment and spread of the most abundant invasive macroalgae in the aquatic environment and that stricter management plans must be implemented to ensure the resilience of the native ecosystem within MPAs.

## 3. Pharmacological Potential of the Invasive Seaweeds

Seaweeds are currently considered a wide source of natural compounds with pharmacological potential [139,194,195,196]. In fact, *Porphyra*/ *Pyropia*/*Neopyropia* spp. was used hundreds of years ago as a food and pharmaceutical source in Asian countries [197]. Moreover, *Pyropia*/*Porphyra/Neopyropia* spp. are known to contain compounds such as porphyran (a sulfated carbohydrate), mycosporine-like amino acids, and phycoerythrin (a phycobiliprotein) with high applicability in the pharmaceutical industry, exhibiting antioxidant [198,199,200], antihypertensive [201,202], anti-inflammatory [203], anticancer [204,205], anticoagulant [199,206], and immunomodulatory [207,208,209,210] bioactivities. 

The agarophyte *A*. *vermiculophyllum* can be, as well, a useful tool in the pharmaceutical industry [211]. Besides agar application in molecular biology techniques and microbiology, the activity of agar as a drug delivery agent has been investigated [212,213]. A recent investigation highlighted the potential of hyaluronic acid-agar-based hydrogels in drug delivery in cases of intravitreal treatments [214].

Aside from their nutritional and nutraceutical properties, *G. turuturu* demonstrated to be a source of bioactive compounds with a pharmaceutical application, namely, R-phycoerythrin [215,216]. Moreover, researchers also tested the antibacterial activity of *G*. *turuturu* harvested in Portugal and they observed that these seaweed polysaccharides at a concentration of 15 mg/mL can significantly inhibit the growth of *Staphylococcus aureus* (88.5%) and *Escherichia coli* (85.4%) [217]. 

Both *A. armata* and *A. taxiformis* have already confirmed to be a pool of bioactive compounds with pharmacological applications. Researchers demonstrated that a concentration of 1 mg/mL of *A. armata* dichloromethane extracts was able to significantly reduce Human colorectal cancer model (Caco‑2 cells) proliferation, proving antitumoral bioactivity [218]. For another perspective, *A. taxiformis* extracts at a concentration of 40 µg/mL exhibited activity against the protozoan *Leishmania infantum*, which affects animals and humans [219]. Therefore, this red seaweed extract also demonstrated to be efficient in the inhibition of fungal growth, namely, *Aspergillus fumigatus*, *Aspergillus terreus,* and *Aspergillus flavus* [220]. Moreover, these seaweed sulfated polysaccharides were found to be antioxidant and non-cytotoxic to HeLa cells [221]. Furthermore, a study revealed that *A. taxiformis* aqueous extract, even in low concentration (1.5%), reveals an antiviral effect on crop plants production due to its phytohormones content, such as cytokinins and auxins [222].

Moreover, the polysaccharide fucogalactoglucan extracted from *C. peregrina* was characterized and its composition revealed to be mainly glucose (50.00–52.91%), galactose (25.5–26.94%), and fucose (20.62–24.56%) [223]. In the same study, its bioactivity was evaluated, demonstrating low cytotoxicity against HeLa cancer cells (< 40%). Besides that, fucogalactoglucan induced RAW264.7 murine macrophage cells to release considerable amounts of nitric oxide (NO) [223]. Thus, the polysaccharide isolated from *C. peregrina* demonstrated to be a possible immunostimulatory agent to improve the human immune system.

Ethanolic extracts of *S. muticum* have proven to reduce significantly the expression of adipogenic marked genes, related to body weight, fat tissue content, serum cholesterol, and triglyceride levels in mice fed with high-fat diet [224]. This assay can be the support to further investigation into possible anti-obesity treatment. The phenolic content shows an interesting antioxidant and anti-inflammatory activity, which can be useful in the food and pharmaceutical industry [225]. Furthermore, *S. muticum* extract revealed bioactivities that protect shrimp against mortal diseases, such as Acute Hepatopancreatic Necrosis Disease [226]. Nevertheless, the current main potential industrial exploitation from *S. muticum* is the alginate extraction; however, in this sector, kelps are preferentially used by the industry due to the higher yields. The alginate extracted from this species can also be used to remove toxic heavy metals from wastewaters or even from organisms in the case of human metals poisoning [227,228,229]. 

*Undaria pinnatifida* has also proven to possess pharmacological potential, likewise anticancer and anti-inflammatory, which can explain the high interest of this seaweed in the food industry worldwide [230]. The application of this Asian kelp as a direct food product acts as a prebiotic, enhancing the intestinal microflora, mainly in the quantity of *Bifidobacterium longum* colony in the human intestine [159].

Regarding the genus *Caulerpa*, it is important to highlight the compound caulerpin, which is an alkaloid that present antitumor [231,232] and anti-inflammatory [233] bioactivity, thus being a potential source for the development of novel marine drugs. However, there are still more pharmacological applications to be found. For instance, a recent study demonstrates that the development of caulerpin-based fish feed can increase lipid content on fish flesh [234]. 

Furthermore, sulfated polysaccharides extracted from the green edible seaweed *C. fragile* has shown antioxidant bioactivity [235].

## 4. Conclusions

The ecological and economic significance of marine algal invasion is undisputed in the global context; however, this topic has not received as much attention as it deserves. Although substantial evidence supports the fact of marine algal introduction and invasion, the underlying ecological principles need more attention to better explain such invasions. 

Marine algal invasions transcend national boundaries, so the problem must be considered an international problem. 

Due to the historical practices of seaweed consumption, the exploitation of invasive seaweeds as a feedstock is a win-win paradigm, economically and as a mitigation control of the invasive macroalgal biomass. Hence, invasive seaweed exhibits a rich nutritional profile in proteins, essential amino acids, lipids, carbohydrates, and minerals that even in low amounts, seaweed direct consumption can be a nutraceutical food product. Moreover, seaweeds are a natural pool of unexplored bioactive compounds with a pharmacological or agricultural application, thus contributing to human health promotion. 

Nevertheless, commercial exploitation of invasive marine algae should be undertaken, if at all, with great care and with a full understanding of all aspects of the biology and ecological consequences of the new exotic species. Despite that, it is necessary to consider that the seaweeds biochemical profile varies according to several biotic and abiotic factors. In this context, it urges the need to chemically characterize them prior to utilization or commercialization. 

## Figures and Tables

**Table 1:** Exotic seaweed species recorded in the Iberian Peninsula. R–Rhodophyta; O–Ochrophyta; C–Chlorophyta.

Phylum	Species	Spain	Portugal	Native Habitat	References
R	*Acrothamnion preissii* (Sonder) E.M. Wollaston 1968	x		Australia	[19]
R	*Agardhiella subulata* (C. Agardh) Kraft and M.J. Wynne 1979	x		Canada	[20]
R	*Agarophyton vermiculophyllum* (Ohmi) Gurgel, J.N. Norris (formerly *Gracilariopsis vermiculophylla* Ohmi et Fredericq 2018)	x	x	Japan	[20,21]
R	*Asparagopsis armata* Harvey 1855	x	x	Australia	[20,22,23]
R	*Asparagopsis taxiformis* (Delile) Trevisan 1845	x	x	Australia	[22,23,24]
R	*Bonnemaisonia hamifera* Hariot 1891	x	x	Japan	[21]
R	*Botryocladia wrightii* (Harvey) W.E. Schmidt, D.L. Ballantine and Fredericq 2017 (formerly *Chrysymenia wrightii* (Harvey) Yamada 1932)	x	x	Japan	[20,21]
R	*Callithamniella flexilis* Baardseth 1941	x		Stoltenhoff Island (South Atlantic)	[20]
R	*Contarinia squamariae* (Meneghini) Denizot 1968 (formerly *Wormskioldia squamariae* Meneghini)		x	-	[21]
R	*Dasya sessilis* Yamada 1928	x	x	Japan	[21]
R	*Dasysiphonia japonica* (Yendo) H.–S. Kim 2012	x		Japan	[21]
R	*Falkenbergia rufolanosa* (Harvey) F. Schmitz 1897	x	x	Australia	[20,25]
R	*Gracilariopsis chorda* (Holmes) Ohmi 1958	x		Japan	[26]
R	*Grateloupia filicina* (J.V. Lamouroux) C. Agardh 1822	x	x	Australia	[21]
R	*Grateloupia subpectinata* Holmes 1912	x	x	Japan	[20,21]
R	*Grateloupia turuturu* Yamada 1941	x	x	Japan	[21]
R	*Gulsonia nodulosa* (Ercegovic) Feldmann and G. Feldmann 1967		x	Adriatic	[21]
R	*Kapraunia schneideri* (Stuercke and Freshwater) A.M. Savoie and G.W.Saunders 2019 (formerly *Polysiphonia schneideri* B. Stuercke and D.W. Freshwater)	x		North America (North Carolina)	[21]
R	*Lomentaria hakodatensis* Yendo 1920	x	x	Japan	[20]
R	*Lophocladia lallemandii* (Montagne) F. Schmitz 1893.	x		Egypt (Suez channel)	[21]
R	*Melanothamnus harveyi* (Bailey) Díaz-Tapia and Maggs 2017 (formerly *Neosiphonia harveyi* (Bailey) M.-S. Kim, H.-G. Choi, Guiry and G.W. Saunders 2001)	x	x	North America (Connecticut)	[20,21]
R	*Pachymeniopsis lanceolata* (K. Okamura) Y. Yamada ex S. Kawabata 1954	x		Japan	[27]
R	*Polysiphonia morrowii* Harvey 1857	x		Japan or Korea	[20]
R	*Pyropia suborbiculata* (Kjellman) J.E. Sutherland, H.G. Choi, M.S. Hwang and W.A. Nelson 2011	x	x	Japan	[20,21]
R	*Scageliopsis patens* E.M. Wollaston 1981	x	x	Australia	[20,21]
R	*Symphyocladia marchantioides* (Harvey) Falkenberg 1897		x	New Zealand	[20,21]
R	*Womersleyella setacea* (Hollenberg) R.E. Norris 1992	x		Hawaii	[21]
O	*Colpomenia peregrina* Sauvageau 1927	x	x	Pacific coast of North America	[23]
O	*Dictyota cyanoloma* Tronholm, De Clerck, A. Gómez-Garreta and Rull Lluch 2010	x		Australia	[19]
O	*Sargassum muticum* (Yendo) Fensholt 1955	x	x	Japan	[21]
O	*Scytosiphon dotyi* M.J. Wynne 1969		x	North America (California)	[21]
O	*Stypopodium schimperi* (Kützing) Verlaque and Boudouresque 1991	x		Egypt (Sinai Peninsula)	[21]
O	*Undaria pinnatifida* (Harvey) Suringar 1873	x	x	Japan	[21]
O	*Zosterocarpus oedogonium* (Meneghini) Bornet 1890		x	Mediterranean	[19]
C	*Caulerpa racemosa* (Forsskål) J. Agardh 1873	x		Australia	[21]
C	*Caulerpa taxifolia* (M. Vahl) C. Agardh 1817	x		India	[19]
C	*Caulerpa cylindracea* Sonder 1845	x		Australia	[19]
C	*Codium arabicum* Kützing 1856		x	Egypt	[19]
C	*Codium fragile* subsp. *fragile* (Suringar) Hariot 1889	x	x	Japan	[21]
C	*Ulva australis* Areschoug 1854	x		Japan	[21]

**Table 2:** *Asparagopsis taxiformis* nutritional characterization from biomass collected in different sampling sites. ND–Non-determined.

Harvesting Site	Lipids	Proteins	Carbohydrates	Ash	Moisture	Reference
Madeira Archipelago (% DW)	6.62	23.76	32.47	N.D.	4.50	[73]
Hawaii (%)	4	6.1	13.2	36	90.03	[74]
Egypt (% DW)	0.4	0.5	17.9	ND	ND	[75]

**Table 3:** Micronutrient and trace element composition of the red seaweeds *G. turuturu*, *A. armata,* and *A. taxiformis* according to Rodrigues et al. (2015), Roque et al. (2019), and Selmi et al. (2020) [70,76,77], and the nutrient value reference (NVR) for each element according to the European Food Safety Authority (ND–Non-determined; ^(a)^ % DW; ^(b)^ mg/day; ^(c)^ mg/kg bw/week).

	Element	Element Concentration (mg/g DW)			NVR (mg)
		*G. turuturu*	*A. armata*	*A. taxiformis*	
Micronutrients	K	20	ND	0.13784	2000
	Mg	2	1.38 ^(a)^	ND	375
	Ca	2.65	4.47 ^(a)^	ND	800
	Na	96.08	9.36 ^(a)^	0.200	600
	P	2.81	0.27 ^(a)^	0.03593	700
Trace elements	Cu	0.01	ND	0.48	1
	Zn	0.07	ND	ND	10
	Fe	0.05	1.188	0.2189	14
	Mn	0.02	0.0623	0.00305	2
	B	0.02	0.0663	ND	10 ^(b)^
	Al	0.01	0.0133	ND	1 ^(c)^
	As	ND	ND	4 × 10^−4^	0.015 ^(c)^
	Cd	ND	ND	2 × 10^−5^	0.007 ^(c)^
	Hg	ND	ND	2 × 10^−5^	0.004 ^(c)^
	Pb	ND	ND	5.1 × 10^−4^	0.005 ^(c)^

**Table 4:** Micronutrients and trace elements in the composition of *Colpomenia peregrina* in South West England (U.K.) according to Beacham et al. (2019) [118] and the nutrient value reference (NVR) for each element according to the European Food Safety Authority ^(a)^ mg/day; ^(b)^ mg/kg bw/week).

Biochemical profile	Element	Concentration (mg/g)	NVR (mg)
Micronutrients	K	46.93	2000
	P	0.67	700
	Ca	55.64	800
Trace Elements	Cu	0.02	1
	Zn	0.05	10
	Sn	0.05	0.055
	Mn	0.23	2
	Si	252.29	20–50 ^(a)^
	Al	19.61	1 ^(b)^
	Fe	9.31	14

**Table 5:** *Sargassum muticum* nutritional characterization from biomass collected in different sampling sites. ND–Non-determined.

Harvesting Site	Lipids	Proteins	Carbohydrates	Ash	Moisture	Reference
U.K. (% DW)	1.6	4.64	69	26.4	ND	[118]
India (% FW)	0.12	5.31	39.25	16.90	87.91	[132]
Spain (% DW)	1.6–3.2	7–22	27.9–44.5	13.2–30.5	ND	[133]
Portugal (% DW)	1.45	16.9	49.3	22.94	9.64	[70]

**Table 6:** Micronutrients and trace elements composition of *Sargassum muticum* harvested in the U.K. [118], India [132], and Spain [133] and the nutrient value reference (NVR) for each element, according to the European Food Safety Authority. ND–Non-dentermined; ^(a)^ mg/kg bw/week.

Biochemical Profile	Element (mg/g)	Country			NVR (mg)
		U.K.	India	Spain	
Micronutrients	K	75.76	13	26.25	2000
	P	1.34	0.4528	ND	700
	Ca	11.2	15.256	47.15	800
	Na	ND	ND	13.75	600
Trace Elements	Fe	0.08	0.537	0.9	14
	Sn	0.06	ND	ND	0.055
	Mn	0.01	0.025	0.03	2
	Al	0.28	ND	ND	1 ^(a)^
	Cu	ND	0.003	0.0155	1
	Zn	0.01	0.128	0.02675	10
	Cr	ND	0.005	ND	0.04
	Mo	ND	0.001	ND	0.05
	I	ND	ND	0.0077	0.15
	Ars	ND	ND	0.0216	0.015 ^(a)^

**Table 7:** *Undaria pinnatifida* nutritional macronutrient characterization from biomass collected in different sampling sites. ND–Not determined.

Harvesting Site	Lipids	Proteins	Carbohydrates	Fibers	Ash	Reference
Japan (Commercial sample % DW)	3.13	14.21	45.08	ND.	37.58	[153]
Japan (% DW)	3.2	15	35.3	2.7	30.8	[154]
Spain (% DW)	1	16.8	37	14.9	28.3	[155]
New Zealand (% DW)	3.30	19.66	50.4	ND	26.58	[150]

**Table 8:** *Undaria pinnatifida* micronutrients and trace elements characterization comparatively to the native and invasive site according to the literature [153,155] and the nutrient value reference (NVR) for each element, according to the European Food Safety Authority. ND–Non-determined; ^(a)^ µg/kg bw/d.

Biochemical Profile	Element (mg/100g DW)	Country		NVR (mg)
		Japan	Spain	
Micronutrients	K	5691	5679	2000
	P	450	1070	700
	Ca	950	693.2	800
	Mg	405	630.2	375
	Na	6494	3511	600
Trace Elements	Mn	0.332	0.69	2
	Ni	0.265	ND	2.8 ^(a)^
	Cu	0.185	0,19	1
	Zn	0.944	3,86	10
	Cr	0.072	ND	0.04
	I	26	9.6	0.15

**Table 9:** Micronutrients and trace elements in the composition of *Caulerpa racemosa* in India, according to Kumar et al. (2001) [170], and the nutrient value reference (NVR) for each element, according to the European Food Safety Authority.

Biochemical Profile	Element	Concentration (% DW)	NVR (mg)
Micronutrients	K	5.03	2000
	Ca	4.76	800
	Mg	4.161	375
	Na	10.64	600
Trace Elements	Fe	29.71	14
	Mn	4.91	2
	Mo	0.15	0.05
	Cu	0.62	1
	Zn	6.82	10
	Se	0.13	0.055
